# The Influence of Recipe Modification and the Technological Method on the Properties of Multigrain Snack Bars

**DOI:** 10.3390/molecules30153160

**Published:** 2025-07-29

**Authors:** Hanna Kowalska, Ewelina Masiarz, Elżbieta Hać-Szymańczuk, Anna Żbikowska, Agata Marzec, Agnieszka Salamon, Mariola Kozłowska, Anna Ignaczak, Małgorzata Chobot, Wioletta Sobocińska, Jolanta Kowalska

**Affiliations:** 1 Department of Food Engineering and Process Management, Institute of Food Sciences, Warsaw University of Life Sciences, 159c Nowoursynowska St., 02-776 Warsaw, Polandagata_marzec@sggw.edu.pl (A.M.); anna_ignaczak@sggw.edu.pl (A.I.); malgorzata_chobot@sggw.edu.pl (M.C.); 2 Department of Biotechnology, Microbiology and Food Evaluation, Division of Food Biotechnology and Microbiology, Faculty of Food Sciences, Warsaw University of Life Sciences, 02-776 Warsaw, Poland; elzbieta_hac-szymanczuk@sggw.edu.pl; 3 Department of Food Assessment and Technology, Institute of Food Science, Warsaw University of Life Sciences, 02-776 Warsaw, Poland; anna_zbikowska@sggw.edu.pl; 4 Institute of Agricultural and Food Biotechnology—State Research Institute, Department of Grain Processing and Bakery, 36 Rakowiecka St., 02-532 Warsaw, Poland; agnieszka.salamon@ibprs.pl; 5 Department of Chemistry, Institute of Food Science, Warsaw University of Life Sciences, 02-776 Warsaw, Poland; 6 Lotte Wedel, 28/30 Zamoyskiego St., 03-801 Warsaw, Poland

**Keywords:** multigrain bars, baking, microwave–vacuum drying, fibre preparation, chemical composition, fat profile, energy value, sensory evaluation

## Abstract

This study aimed to assess the use of selected raw materials, such as whole-grain oat flakes, pumpkin seeds, sunflower seeds, and flaxseeds, to obtain bars using baking and drying methods. Modifying the bars’ composition involved selecting the fibre preparation, replacing water with NFC juice, and using fresh apple juice and apple pomace. The Psyllium fibre preparation, also in the form of a mixture with apple fibre, was the most useful in dough cohesion and the quality of the bars. Baked bars were characterised by higher sensory quality than those obtained by drying. Microwave–convection drying was a good alternative to baking, primarily due to the lower temperature resulting in a lower acrylamide content and comparable product quality. The basic grain ingredients and fibre preparations mainly shaped the nutritional and energy value and the sensory and microbiological quality. Modifying the recipe using NFC or fresh juice and apple pomace allowed the bars to develop new properties and quality characteristics. The use of NFC juices resulted in a reduction in the pH of the bars, which is associated with a higher microbiological quality of the bars. All bars had low acrylamide content, significantly lower than the permissible level. Using fresh pomace or fibre preparations made from by-products is a possibility to increase the fibre content in the bars and a method of managing by-products.

## 1. Introduction

Multigrain cereal bars are ready-to-eat products classified as snacks. Depending on the type, snacks outside of main meals can provide energy and health-promoting ingredients, which may lead to the consumption of smaller portions of traditional meals. Conscious, sensible “snacking”, especially of recommended products, has a beneficial effect on the body, without exposing it to “hunger stress”. Consumption of snacks can become a form of diet diversification, providing the necessary ingredients, and even pre- and probiotics [1]. The so-called “healthy snack” concept appears in the media and many publications, and is determined by nutrient density. Minimally processed products are recommended, containing nutrients and health-promoting ingredients, without added simple sugars, with an unfavourable fatty acid profile, and so-called “artificial” additives. Klerks et al. [2], researching the cereal bar market in Germany, indicated the need to increase the nutritional quality of bars and their naturalness index. Souiy et al. [3] used the naturalness index for gluten and sugar-free cereal bars with spirulina, taking into account the number of additives, agricultural practices, the degree of processing of ingredients, and the number of unnecessary ingredients. According to Konopacka et al. [4], the nutritional importance of tissue raw materials is greater than previously thought. Anthocyanins consumed with a natural fruit matrix are more beneficial than those isolated and consumed in dietary supplements. Similarly, the composition of multigrain bars, i.e., cereal ingredients, seeds, nuts, fruits, and vegetables, is a source of complex polysaccharides, fats, proteins, vitamins, minerals, fibre, and many other components naturally associated with the plant matrix [5,6], also including insect protein [7,8], that determine the quality of the product, both in terms of health-promoting and sensory properties, as well as sustainable food production techniques. Due to their unique nutritional profile, oat products containing β-glucan (a natural component of the soluble fraction of dietary fibre) are beneficial for health [9].

An important aspect in the production of multigrain bars is the selection of the recipe for ingredients, including those that play a binding role. According to nutritional trends, snacks should be processed as little as possible to shape the high nutritional and functional value of the products [5,6,10,11]. Composing bars with the addition of jackfruit seed flour [12], sesame bars enriched with red beetroot and ginger or fruits, pumpkin bars [13], multigrain bars with seeds, vegetables, fruits, and nuts, as well as herbs or spices (black cumin, saffron) [14], with mung beans [15] and other plant raw materials, including by-products [16], may create great opportunities for the production of bars with the desired nutritional and health-promoting composition. Budin et al. [17] increased the content of bioactive compounds in cereal bars by adding yerba mate extract. Water and fat contained in seeds such as sunflower, pumpkin, and linseed have a beneficial effect on the preparation and formation of dough for bars, and crushed dates are used as a binder for various bars [6,15,18]. In a study by Kowalska et al. [5], blended fresh kale was used to produce multigrain bars. Many researchers [19,20,21,22] used lecithin to produce cereal bars. Other researchers used pectin [23], guar gum [24], xanthan gum [25], or acacia gum [26]. Melati et al. [27] also used gums as a binder for salty cereal bars, as well as collagen and Psyllium. Honey, malt extract, and concentrated juices can also play a binding role and shape sensory and nutritional values of food [6,18,28]. Timm et al. 2020 [29] used nanosuspensions from the seed coat of pinhao (*Araucaria angustifolia*) as binders for cereal bars, and Mirpoor et al. [30] used by-products from various seeds after oil extraction, rich in fibre, proteins, and secondary metabolites. In bar production technology, binding ingredients influence the formation of the appropriate consistency of bar dough and the texture of the finished bars. In addition to being an important source of fibre in the human diet, fibre preparations perform technological functions, are important in binding ingredients, and create the structure of bars [31]. The use of NFC juices as binding ingredients, discussed in an earlier publication [6], has not yet been reflected in the wider literature.

Bars can be manufactured using various methods. The simplest method involves composing loose ingredients and combining them with appropriately prepared liquid sugar syrup ingredients, mixing, forming, stabilising, and packaging. Extrusion and baking are frequently used methods [11], but drying methods are also used [32]. Depending on the bar recipe, various procedures are used as production stages, such as pulping, homogenisation, or only partial grinding, heating, mixing, and cooling, as well as thermal processing (extrusion, baking, drying) [6,19,32]. There is a lack of information on the selection of the composition of a recipe without added sugar or sweeteners and other highly processed ingredients, and the techniques of manufacturing bars using baking and drying in the context of shaping their texture and sensory quality, as well as nutritional and microbiological value. In cereal bars, there is a risk of contamination with mycotoxins and the occurrence of acrylamide, polycyclic aromatic hydrocarbons (PAHs), or 5-hydroxymethylfurfural (HMF), which may adversely affect the health of the consumer [33,34].

This study aimed to demonstrate the impact of modifying the recipe for bars by adding fibre preparations, NFC juices, and heat treatment (baking, drying) on their physico-chemical properties, health-promoting potential, and sensory and microbiological quality. An 80 ± 2 g bar recipe was developed using water (30%; g/100 g of raw material) and ingredients such as whole-grain rolled oats (14%), pumpkin seeds (10%) and sunflower seeds (10%), flaxseed (10%), Psyllium fibre preparation (6%), and barley malt extract (20%). The research also justified the selection of the multigrain bar recipe and its modification by adding fresh apple juice and pomace.

## 2. Results and Discussion

The selection of the bar recipe resulted from the growing demand for products that are an alternative to highly processed, conventional sweet and salty snacks. The technological conditions for producing easy-to-form bar dough were taken into account. An appropriate sensory quality of the bars was also desired, hence only partial grinding of the grainy ingredients to emphasise the naturalness of the product [35]. A recipe for bars was designed based on water (30%; g/100 g raw materials) and raw materials such as whole-grain oat flakes (14%), pumpkin (10%) and sunflower seeds (10%), linseed (10%), Psyllium fibre preparation (6%), and barley malt extract (20%). Such ingredients provide an important source of essential fatty acids (EFAs) and protein, as well as complex carbohydrates and other components that contribute to antioxidant activity. An important ingredient was dietary fibre added in the form of commercial preparations, usually obtained from pomace, as well as NFC juices, which, in addition to natural ingredients with antioxidant properties, acted as a binder for the bar dough and allowed the bars to be formed before heat treatment.

The scope of the research included the assessment of the addition of fibre preparations and NFC juices on the properties of the bars. Additionally, modifications to the recipe using pomace and fresh apple juice were tested in a narrower scope. The thermal treatment of the bars consisted of baking or microwave–convection drying of samples formed in the shape of bars.

### 2.1. Evaluation of the Effect of the Addition of a Fibre Preparation and NFC Juices on the Physical Properties of Baked and Dried Bars

#### 2.1.1. Water Content and Activity, pH, and Colour

**Figure 1:**
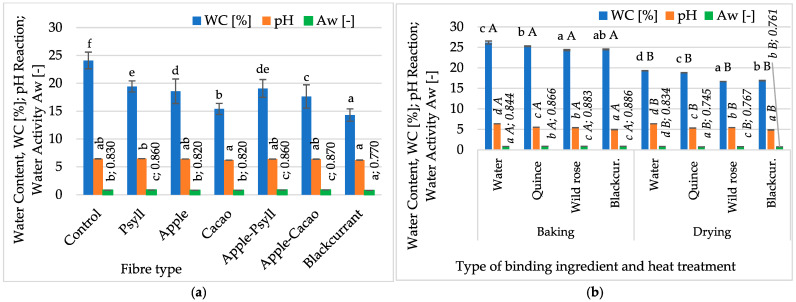
Changes in water content, pH, and water activity of bars, depending on (**a**) fibre preparation of bars without NFC juice; (**b**) binding type (NFC juices) and thermal treatment (baking, drying) of bars with Psyllium fibre. Designations a, b, c, d, e, and f—the effect of the type of fibre preparation; *a*, *b*, *c*, and *d*—the impact of binding type (NFC juice); and *A* and *B*—the impact of thermal treatment.

To investigate the effect of the fibre preparation on the water content and activity in oven-baked (180 °C, 25 min) multigrain bars, single-fibre formulations or two-component mixtures were used. An apparent, statistically significant effect of fibre on the water content (WC) and water activity (Aw) in the bars was demonstrated, but a much lower impact on the pH value was observed (Figure 1a). The highest WC, about 24.1%, was found in control samples, without added fibre, with an Aw of about 0.83. The addition of fibre preparations in powder form caused a lower water content by 5–10% but smaller changes in the water activity value, at a level of 0.77–0.87. Also, samples containing apple fibre or its mixtures with cocoa fibre or Psyllium were characterised by relatively high moisture (17.6–19.4%) and water activity (0.82–0.87).

The share of Psyllium and Apple–Cacao fibre caused a slight increase in Aw, up to 0.87, and a decrease in the remaining bars, with the lowest value obtained when using blackcurrant fibre (0.77). Higher values of these indicators in bars with Psyllium and apple fibres may result from their ability to bind water, simultaneously shaping the texture of the bars. Too low a water content could be associated with undesirable hardness of the bars. On the other hand, the obtained water activity values indicate a limited shelf life of the bars and possible susceptibility to microbiological changes during storage. The relatively high water activity of the bars may be the result of baking, the aim of which is not to reduce the activity and water content, but to obtain a product with increased digestibility and assimilability as well as sensory quality.

The goal of manufacturing bars focused on obtaining a snack product with a shelf life of up to 14 days. All bars were characterised by relatively high water activity values, but below the range in which pathogenic microorganisms can grow. It can be assumed that properly packaged bars can be stable within the indicated storage period. In the studies of Safvi et al. [36], the moisture content of baked cereal bars (180 °C/30 min) with chironji seeds and condensed milk was in the range of 8.0–13.3. A high-energy cereal bar with a recipe of min. 60% cereals, min. 14% dried fruits, and min. 20% sweeteners, produced by mixing the ingredients with heated corn syrup and honey, showed a lower water activity of 0.37–0.50 [37].

Psyllium fibre was used in the next series of bars, and the effect of replacing water as a binding component with NFC juices from wild rose, quince, and blackcurrant was assessed (Figure 1b). The juices led to the obtaining of a higher water content and activity of bars with Psyllium fibre. Samples baked in the oven had significantly higher water content and activity, 24.3–28.2% and 0.87–0.89, respectively, than samples obtained by microwave–convection drying, 16.7–19.3% and 0.74–0.83, respectively. WC values of bars baked with the use of fibres were significantly lower, in the range of 14.3–24.1% (Figure 1a), compared to the control sample (24.1%) and bars in which NFC juices (14.3–19.4%) were used as binding components (Figure 1b). This may be due to the properties of the raw materials and the environmental conditions (temperature, air humidity). Juices replacing water in a 1:1 ratio contained dissolved ingredients (12–18 °Bx), which is why they reduced the moisture content of the bars.

The pH values of the bars showed slight differences, ranging from 6.22 to 6.49 (Figure 1a). The use of currant or cocoa fibre resulted in a slightly lower pH (6.22 to 6.23), and Psyllium fibre resulted in a higher pH (6.49). Safvi et al. [36] found a relatively narrow pH range in energy bars, 5.26 to 5.53, and slight changes were observed in the share of chironji seeds and condensed milk. The type of binder in the bar formula and the method of its production (heat treatment) had a significant effect on the pH (Figure 1b). Replacing water with NFC juices in baked and dried bars reduced the pH by 0.8 to 1.5. NFC blackcurrant juice reduced the pH of baked and dried bars to 4.86–4.96, while with water, the pH was about 6.36. Smaller differences, 5.32–5.54, characterised the pH of dried and baked bars with quince juice. The reduction in moisture and pH of the bars allows us to assume an increase in their microbiological safety and, consequently, the possibility of extending their shelf life. Lower pH is also associated with a better preservation of some nutrients and colours. This confirms the possibility of using NFC juices to produce multigrain bars as enriching ingredients that perform a technological function.

**Figure 2:**
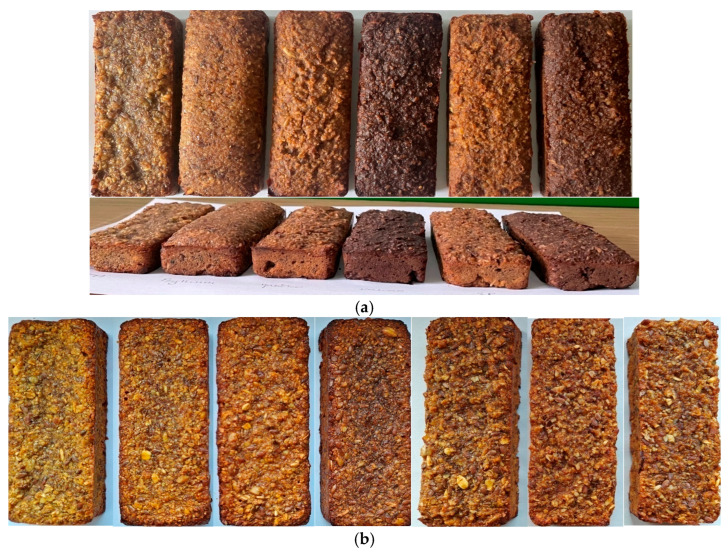
Photos of baked bars: (**a**) with fibre preparation, from left to right—control (no fibre), Psyllium, apple, cocoa, Apple–Psyllium, and Apple–Cocoa; (**b**) with Psyllium fibre, from left to right—water, quince, wild rose, and blackcurrant NFC juices, then with apple pomace in the amounts of 2, 6, and 12%.

**Figure 3:**
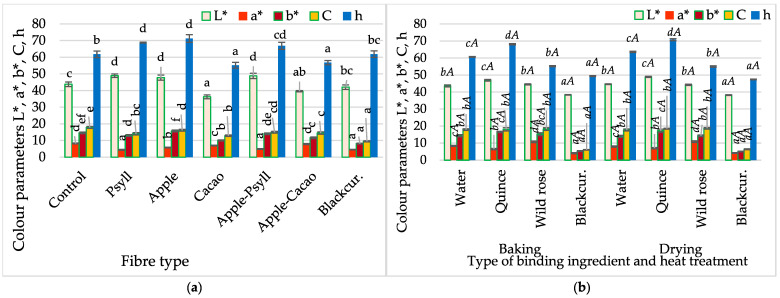
Changes in colour parameters of bars, depending on (**a**) fibre preparation of bars without NFC juice; (**b**) binding type (quince, wild rose, blackcurrant NFC juices) and thermal treatment (baking, drying) of bars with Psyllium fibre. Designations a, b, c, d, e, and f—the effect of the type of fibre preparation; *a*, *b*, *c*, and *d*—the impact of binding type (NFC juice); and *A*—the impact of thermal treatment.

The colour of the bars depended on the specific colour of the fibre and NFC juices (Figure 2 and Figure 3). Compared with the colour of the control bars (without fibre and NFC juice) determined based on the colour parameters L*, a*, and b*, the absolute colour difference ranged from 4.7 to 8.8. The most significant differences in the colour of the bars with cocoa and blackcurrant fibre were related to the darkening of their colour, and in the bars with apple and Psyllium fibre, to the increased lightness of their colour. The heat treatment method (baking, drying) had no significant effect on the colour changes in the bars.

#### 2.1.2. Texture of Bars

**Figure 4:**
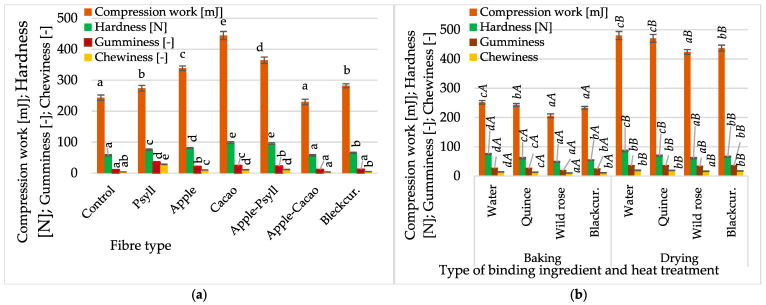
Changes in compression work, hardness, gumminess, and chewiness, depending on (**a**) fibre preparation of bars without NFC juice; (**b**) binding type (quince, wild rose, blackcurrant NFC juices) and thermal treatment (baking, drying) of bars with Psyllium fibre. Designations a, b, c, d, and e—the effect of the type of fibre preparation; *a*, *b*, *c*, and *d*—the impact of binding type (NFC juice); and *A* and *B*—the impact of thermal treatment.

In the case of baked bars, a lower hardness and a crispy crust are desirable. Partially crushed grainy ingredients shaped the specific texture of the bars. Compared to bars without fibre (control), fibre type significantly influenced the texture profile of baked bars by increasing values for hardness and work of compression, as well as gumminess and chewiness in most samples (Figure 4).

The highest hardness and compression work of bars with cocoa fibre were associated with low gumminess, contrary to bars with Psyllium fibre, which were the most gummy. The other bars were quite hard and had less gumminess, except those with Apple–Cacao, which had lower compression work (Figure 4a). The chewiness values of bars with Psyllium were about 2.5–2.7 times lower than those with apple fibre, Apple–Psyll, and Apple–Cacao, and the subsequent indices were 6–7 times lower.

Dietary fibre, especially the Psyllium fibre preparation, plays a very useful technological role in bar production. Compared to using other fibres and control bars, this fibre resulted in significantly lower hardness and greater gumminess and chewiness. Compared to using apple fibre and other fibres alone, using a mixture of preparations with Psyllium fibre (Apple–Psyll) was of great importance for shaping the texture of the bars, which were firm and did not crumble. The use of apple fibre, the Apple–Psyll mixture, and cocoa can also be considered in the design of the bar recipe. By showing comparable gumminess, although significantly lower compared to Psyllium fibre, these fibres can be very useful in managing waste products from plant raw materials and thus contribute to sustainable technologies.

As demonstrated by Nawirska and Kwaśniewska [38], fibre affects the texture of food, as well as the durability of the product, which is the result of its ability to thicken, impart viscosity, and retain water, as well as fat. It is also associated with reduced mass loss during heat treatment and increased production efficiency. These features turned out to be desirable in the case of the tested baked bars. The selection of ingredients shaping the texture of bars is not easy and obvious. Iuliano et al. [39] evaluated the beneficial effect of grated coconut and honey in the development of a quinoa–amaranth cereal bar, but also the negative impact of increasing puffed and flaked quinoa, while the binder agar-agar negatively affected texture as well as taste and sweetness attributes.

The texture of the bars was less affected by the type of binding component, which was water or NFC juices, than by heat treatment (Figure 4b). In baked bars, NFC juices caused lower hardness and work compression than the addition of fibres (Figure 4a,b). Compared to baking, using microwave–vacuum drying of bars with juice caused a significant increase in the texture profile indices of the bars. Bag compression values of dried bars were about two times higher (480.1–424.1 mJ) than those of baked bars (205.8–252.0 N) (Figure 4b). Convection–microwave drying caused an increase in the hardness of the bars in the range from 59.8 to 84.2 N. The hardness of baked bars was lower by 12–20%. The lowest hardness was shown by bars with wild rose juice (48.9–61.0 N). Gumminess and chewiness of dried bars were 23–43% and 27–35% higher than those of baked bars, respectively. Since dried bars were harder, it may be justified to use a lower thickness to make the product more breakable and crunchy [6]. In a study by Souiy et al. [3], the hardness of the bars was much lower, only about 0.5 N.

Compression work and hardness of bars with NFC juice were 2–25% and 2–18% lower, respectively, than those without juice (Figure 4b). The most significant reduction in the values of both indices was observed in bars with wild rose juice, and the smallest in bars with quince juice. Bars baked and dried with juice showed lower hardness values (48.9–60.9 N) than bars without juice (75.1–85.8 N). The type of binder had no significant effect on gumminess and chewiness of the bars. Regardless of the method of obtaining bars, only a slight increase in chewiness was observed, according to the order of using rose juice, blackcurrant, quince, and water. Significant negative water content and activity correlations were found with all TPA indices (from −0.720 to −0.879) except for hardness, which positively correlated only with chewiness. Reducing the water content and activity increased the compression work, hardness, gumminess, and chewiness, but decreased cohesiveness (data not presented). Due to the effect of NFC juices, which is comparable to that of the use of water as a binding component, their use may find practical application in producing bars on a larger scale. Moreover, using selected drying methods to obtain multigrain bars in addition to traditional baking seems realistic.

### 2.2. Evaluation of the Addition of Fibre Preparation and NFC Juices on the Chemical Properties of Baked and Dried Bars

#### 2.2.1. Acrylamide Content in Bars

**Figure 5:**
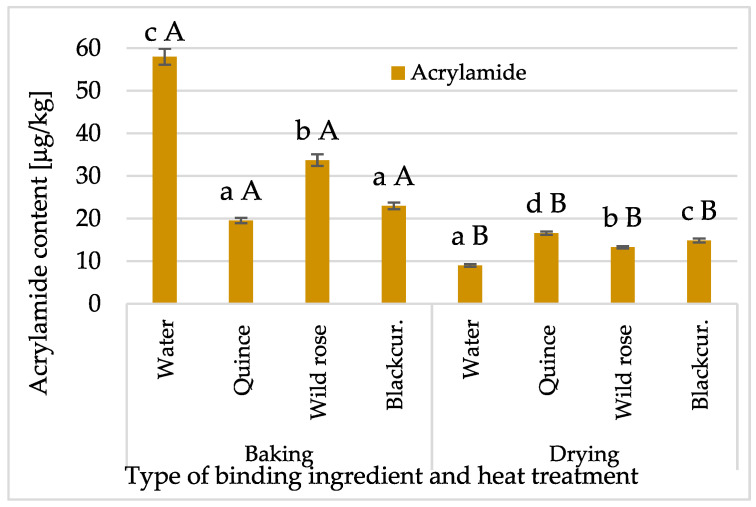
Changes in acrylamide content, depending on binding type (quince, wild rose, blackcurrant NFC juices) and thermal treatment (baking, drying) of bars with Psyllium fibre. Designations a, b, c, and d—the impact of binding type (NFC juice); and A and B—the impact of thermal treatment.

Acrylamide content was tested in baked and dried bars with Psyllium fibre and NFC juices. The obtained results were many times lower than the permissible level of acrylamide in a food product, i.e., 300 µg/kg, established in the Regulation of the European Commission 2017/2158/EC [40]. A significant effect of the binding component and the method of obtaining the bars on the acrylamide content was found (Figure 5). The bars obtained by the baking method had a higher acrylamide content (33.5 µg/kg) due to the baking conditions, i.e., the use of a temperature of 180 °C for 25 min. The lower contents of this compound in dried bars (on average 13.6 µg/kg) resulted mainly from using a lower temperature (40–80 °C) for 25–45 min.

The highest acrylamide content was recorded in control samples, namely, baked bars (average 57.0 µg/kg), and the lowest in control samples obtained by microwave–convective drying (average 8.9 µg/kg). Žilić et al. [41] examined acrylamide content in various commercial corn-based snacks. They showed that its content ranged from 1.9 to 799 ng/g, including approximately 37% of products exceeding the level for products based on whole-grain cereals, specified in the European Commission Regulation 2017/2158/EC [40].

It has been shown that high amounts of acrylamide are formed during food processing at temperatures of 100–120 °C and higher [42]. Becalski et al. [43] reported that despite temperatures below 100 °C, significant amounts of acrylamide were formed in dried fruits and juices, which was associated with an extended drying time. They also found that the increased acrylamide content in dried plum products may be due to asparagine and sugars. At elevated temperatures, free amino acids and reducing sugars present in food are the main precursors of the Maillard reaction, generating many sensory active substances that play a key role in the development of aroma, colour, and taste [44]. Acrylamide formed in the Maillard reaction and present in food is considered mutagenic or carcinogenic to humans [45]. The primary precursor of acrylamide in food is free asparagine [46], which can be converted into acrylamide via decarboxylation and deamination reactions under thermal conditions, and its production is increased in the presence of carbonyl groups [47]. Cereal-based foods are one of the diet’s main sources of Maillard reaction products. Žilić et al. [48] analysed the content of sugars and free asparagine to determine the potential of ten cereal varieties to form acrylamide during heat treatment. They found the highest glucose, fructose, and asparagine contents in rye and oat varieties. Therefore, whole-grain oat flakes, the basic ingredient of the bars studied, and juices containing sugars can be considered the primary source of compounds that are precursors in forming acrylamide during heat treatment.

#### 2.2.2. Polyphenol Content and Antioxidant Activity

**Figure 6:**
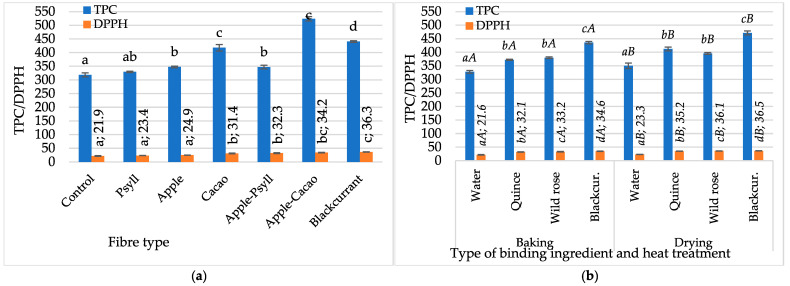
Changes in total polyphenol content, TPC [mg GAE/100 g d.m.], and antioxidant activity, DPPH [µM Trolox/100 g d.m.], depending on (**a**) fibre preparation of baked bars without NFC juice; (**b**) binding type (NFC juices) and thermal treatment of bars with Psyllium fibre. Designations a, b, c, and d—the effect of the type of fibre preparation; *a*, *b*, *c*, and *d*—the impact of binding type (NFC juice); and *A* and *B*—the impact of thermal treatment.

One of the most important goals of adding NFC juices to the bar formula was to enrich them with natural bioactive ingredients. The use of juices as enrichment ingredients is a response to the recommendations to increase the share of fruit and vegetables in the diet [49], which can be consumed in various forms. Baked control bars (without fibre) and with Psyllium fibre without juice (with water) were characterised by a total polyphenol content of about 318.5 mg GAE/100 g d.m. and antioxidant activity of about 21.9 µM Trolox/100 g d.m., and dried bars by 350.0 mg GAE/100 g d.m. (TPC) and 23.3 µM Trolox/100 g d.m. (DPPH) (Figure 6a,b). The use of NFC juices and fibre preparations significantly enriched the bars (*p* < 0.05) in polyphenolic compounds (by 19–64%) and increased antioxidant activity (by 6–66%). A greater effect of fibres than juices was found in increasing the TPC value, but a greater effect of juices on DPPH values was observed. Compared to bars without fibre and juices, in bars with fibres, TPC and DPPH increased by 6–39% and 6–40%, respectively, and in bars with juices by 12–30% and 33–41%. Compared with baked bars, significantly higher TPC values and lower DPPH values were found in dried bars. The highest total polyphenol (TPC) contents were found in bars with the Apple–Cacao fibre mixture (523.8 mg GAE/100 g d.m.), and in terms of the use of NFC juices, in bars with the addition of blackcurrant juice (435.4–471.4 mg GAE/100 g d.m.).

The content of polyphenolic compounds and antioxidant activity of the analysed bars result, among others, from the high bioactive potential of the ingredients used to obtain them, i.e., pumpkin and sunflower seeds, linseed, whole-grain oat flakes, fibre preparations, and NFC juices [6,50,51].

Fibre preparations produced from fruit pomace and cocoa husk are a source of polyphenols [52,53]. Wild rose fruits [54], quince [55,56], and blackcurrant [57], and therefore NFC juices obtained from them, are a valuable source of many bioactive compounds, especially natural antioxidants, such as vitamin C, polyphenols, carotenoids, tocopherols, and polysaccharides. Compounds with antioxidant properties prevent many diseases and thus fit into the trend of the so-called “healthy lifestyle” [58]. From a technological perspective, NFC juices can replace water, facilitate shaping before heat treatment, shape sensory values, and enhance the product’s durability. The selection of raw materials and the method of manufacturing multigrain snacks can affect health maintenance, such as polyphenols, carotenoids [59], and other antioxidants, as well as their bioavailability and associated benefits [60]. According to Stankiewicz and Wieczorkiewicz [61], the presence of anthocyanins in food is of great importance because their small share protects ascorbic acid against oxidation, even during high-temperature processing. And Souiy et al. [3] showed that the addition of 2% spirulina in cereal bars increased the TPC content by 2–3 times, from 32 to 102 mg GAE/100 g.

#### 2.2.3. Nutritional Content of Selected Bars

In selected baked bars with the addition of apple juice and pomace, macro- and micronutrients were determined, comparing their content to bars with Psyllium fibre (Table 1), and the fat profile of bars with fibres and NFC juice was examined (Table 2). The share of fresh juice and apple pomace in the bar recipe caused a decrease in the content of nutrients, but an increase in the content of total dietary fibre and its fraction insoluble in water (Table 1).

The 12% share of pomace reduced the fat content by about 5%, protein by about 1.5%, and minerals by about 0.5%. These differences result from adding apple pomace, which contains negligible amounts of fat and protein, but significant amounts of fibre, especially soluble in water. Therefore, the total fibre content increased by about 1%, including the soluble fraction by more than two times. The high dietary fibre content in the bars is one of the most important advantages of these products, both in terms of health-promoting and technological values. However, neither its deficiency nor its excess in the body is beneficial. Too much fibre reduces the absorption of minerals and fats and, consequently, fat-soluble vitamins. The insoluble fraction is made up of cellulose, mostly hemicellulose, and lignin, while the soluble fraction includes beta-glucans, pectins, fructooligosaccharides, resistant starch, plant gums (e.g., guar gum), plant mucilages (e.g., Psyllium), and some hemicelluloses [62]. Soluble fibre is fermented in the large intestine [63]. As a result of fermentation, short-chain fatty acids (acetic, propionic, butyric) are formed, which lower the pH in the lumen of the large intestine and favourably condition the development of lactic acid bacteria and maintain the appropriate proportion between probiotic and putrefactive bacteria. Naveed et al. [64] proved that increased dietary fibre promotes satiety, aids weight management, and prevents obesity.

The bars were characterised by high fat content, in the range of 21.9–26.6%. Increasing the share of apple pomace resulted in a lower fat content (Table 1). In Boukid et al.’s [65] studies, amaranth-based multigrain bars showed significant nutritional benefits, especially the protein content, which reached 21.95%, about 40% higher than in traditional bars. Safvi et al. [36] showed that the fat, protein, and ash contents in baked bars based on oat grain, puffed rice, chironji seeds, almonds, dates, cane sugar jaggery, raisins, and condensed milk were in the ranges of 13.3–28.7%, 8.2–12.9%, and 1.7–4.7%, respectively.

The bars contained significant amounts of unsaturated fatty acids within relatively narrow ranges, in total, 13.56–14.42% (Table 2). From a nutritional point of view, this is very beneficial because these acids can counteract the development of circulatory system diseases [66]. The main group of fatty acids (approximately 85.6%) in the tested samples was linoleic, oleic, and γ-linolenic acids, i.e., omega acids with numerous positive functions in the human body. The largest share in the bars was linoleic acid (Table 2), belonging to the group of exogenous omega-6 fatty acids, used in the prevention and treatment of diseases, including circulatory system diseases, diabetes, osteoporosis, and cancers [66]. For comparison, Derewiaka and Górska [67] showed that the fat profile of selected cereal bars available on the market is high in saturated fatty acids. It is not recommended to reduce the share of energy from total fat below the recommended values of 20–35% of energy demand [68,69] due to the risk of insufficient intake of EFAs and fat-soluble vitamins.

Bars with Psyllium fibre, apple pomace, and juice were characterised by a relatively high content of carbohydrates (approximately 30.1–31.2%) and protein (15.2–16.7%). No significant effect of fresh apple juice and pomace on the content of carbohydrates and protein was demonstrated. Carbohydrates constitute a large group of organic compounds; in terms of nutrition, they are digestible and non-digestible. Complex carbohydrates, oligo- or polysaccharides, are more desirable in the diet. Digestible carbohydrates are the primary energy source for complete oxidation in the Krebs cycle. With their insufficient supply, fatty acids are not completely burned, so ketone bodies are formed, which acidify the body. Both long-term deficiency and excess of carbohydrates in the diet are detrimental to health [62]. Cereal bars are a valuable source of protein, the basic nutritional component of the human diet. The properties and functions of proteins depend on their structure [70]. Technological processes occurring during baking, e.g., Maillard reactions, can affect the content of individual amino acids and the mutual proportions between them. These reactions reduce, among others, the amount available of the amino acid lysine.

The addition of apple pomace significantly reduced total polyphenols, reaching 24.5%, compared to bars with Psyllium fibre (Table 1). On the other hand, antioxidant activity, at a level of 23.06 μM Trolox/100 g d.m. in the base bars, increased almost 4-fold after adding apple pomace. This could result from the increased content of compounds exhibiting antioxidant potential and the formation of various compounds at the high temperature of baking the bars. Oxidation, denaturation, and hydrolysis reactions could occur, including Maillard and caramelisation reactions. The products of these reactions shape structural, sorption, and sensory properties, ensuring the overall attractiveness of the product [71], and can also increase antioxidant activity and bioavailability [60].

Cereal and by-product products, especially pomace and bran as sources of fibre, in the modern food industry reveal many opportunities to improve global health, environmental sustainability, and responsible consumption [72]. Recommendations related to increasing fibre intake are considered to be a factor reducing the risk of many diseases [1]. Souiy et al. [3] showed that the nutritional quality of cereal bars can be assessed by calculating a Nutri-Score using the Food Standards Agency’s Nutrient Profiling System (FSA-NPS) and the degree of naturalness. Multigrain cereal bars are not only a convenient snack, but also a nutritious product with a rich nutritional profile, which consists of carbohydrates, fat, proteins, fibre, and essential vitamins [73]. Whole-grain oat flakes, pumpkin seeds, sunflower seeds, and linseeds are a good source of minerals [51,74].

The energy value (calorie content) of selected bars was also assessed, which depends on the content of nutrients such as fats, carbohydrates, including sugars, and protein. The calorific value of the bars was within a relatively narrow range, from 289 to 309 kcal/100 g (Table 3). Bars with NFC juices and bars with the addition of NFC juices and a mixture of apple and cocoa fibres had a higher calorific value than those without fibre and water. In relation to similar bar snacks available on the market, the calorific value of the developed bars did not exceed the average calorific value for this type of product.

A higher energy value (447–475 kcal/100 g) was found by Olagunju et al. [11] in baked (150 °C/30 min) cereal bars based on pre-fermented amaranth flour, acha, and pearl millet.

### 2.3. The Influence of Fibre and the Binder on the Sensory Quality of Baked and Dried Bars

**Figure 7:**
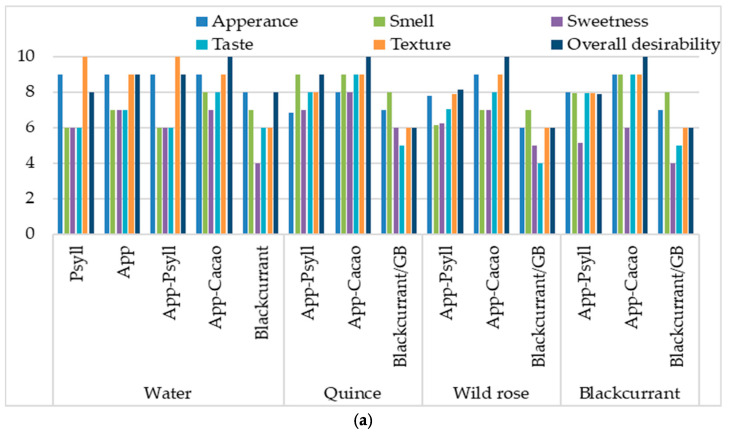

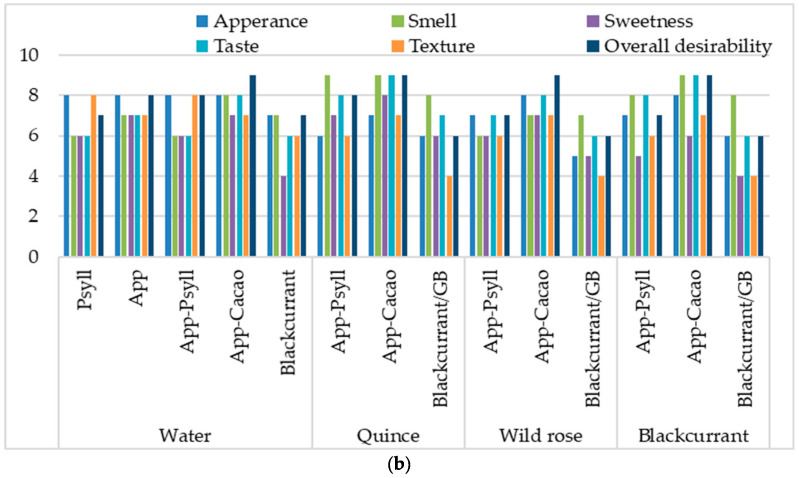
The influence of fibre preparation and binding ingredients on the sensory quality of dried bars, depending on thermal treatment: (**a**) baking; (**b**) microwave–convection drying.

The sensory quality of the bars obtained by baking and microwave–convection drying was assessed positively (Figure 7). The addition of fibre positively affected the overall appearance and texture of the bars (Figure 7a). The structure of the bars was relatively compact and uniform. On a 10-point scale, the baked samples were rated higher, ranging from 6.1 points for the bars with blackcurrant fibre and NFC rose and blackcurrant juices, to 8.7 points for the bars with the Apple–Cacao fibre mixture. The same samples obtained by the drying method were rated from 5.6 to 8.1 points (Figure 7b). The taste attribute defined as “sweetness” influenced the overall quality, which was awarded 4–5 points. The highest-rated characteristics were texture and general desirability, for which 8–10 points were awarded in the baked bars. In the case of dried bars, the external appearance, texture, and overall desirability were rated highly, most often at around 8 points, especially when Psyllium fibre or its mixture with apple fibre was used. A harder crust and soft crumbs characterised the more desirable baked bars, and those obtained by the drying method were harder than those obtained in the oven, without showing softer crumbs.

When comparing bars containing different fibre preparations, the panellists rated the samples containing a mixture of apple and cocoa fibres the best, which provided a characteristic, attractive appearance, taste, smell, and colour. Even higher scores were given for appearance, colour, and overall desirability when NFC juices were added to the bars with the mixture of these fibres. Regarding texture and overall desirability, bars with Psyllium fibre or the Apple–Psyll mixture were rated the highest. Regardless of the type of fibre, bars containing wild rose juice were rated lower because they did not provide a characteristic taste, smell, and colour. Bars containing quince juice were rated highly due to their sweetness, interesting smell, and taste. In the case of blackcurrant juice, due to its high acidity, bars containing it were rated the lowest. However, they received high scores due to their unique aroma, characteristic taste, and colour, which increased overall desirability.

Considering the overall acceptability and nutritional value, bars with natural ingredients (fibres, NFC juices) were rated highly. In other studies, sensory evaluation showed the acceptability of high-energy cereal bars with a gross energy value of 481–680 kcal/100 g [37]. Gluten-free and sugar-free cereal bars enriched with spirulina and flavoured with neroli essential oil obtained by drying (25 °C) of combined granular and liquid ingredients were also rated highly (6.8–8.0 out of 9.0 points) in terms of hardness, crispness, cohesiveness, chewiness, gumminess, taste, and odour [3].

### 2.4. Microbiological Quality of Selected Bars After Production and Storage

Before the bars were manufactured, the microbiological quality of the raw materials used for their production was analysed. No Staphylococci or *Enterobacteriaceae* bacteria were detected in any of the raw materials. Additionally, NFC juices were free of mesophilic aerobic microorganisms, spore-forming bacteria, and fungi, while the remaining raw materials were characterised by low contamination with these groups of microorganisms. The highest number of mesophilic aerobic microorganisms was detected in linseed and pumpkin seeds (9.8 × 10^3^ and 5.3 × 10^3^ cfu/g, respectively). The number of spore-forming bacteria ranged from 4.1 × 10^1^ cfu/g (oat flakes) to 9.7 × 10^2^ cfu/g (barley malt). Contamination of raw materials with fungi ranged from 2.0 × 10^2^ cfu/g (sunflower seeds) to 5.1 × 10^2^ cfu/g (pumpkin seeds). Changes in the microbiological quality of baked bars with selected fibres and NFC juices were assessed (Table 4).

In all bar samples, as in the raw materials, no Staphylococci or *Enterobacteriaceae* bacteria were detected. In the bar samples taken directly after production, no yeasts or moulds were detected; however, after 7 days of storage at room temperature (approx. 22 °C), they were not detected in only two types of bars: those with quince and wild rose juice, to which blackcurrant fibre was added. After 7 days of storage, the highest number of these microorganisms, 2.3–2.5 × 10^4^ cfu/g, was found in bars with Psyllium fibre and quince and wild rose juices. After 14 days of storage, the number of yeasts and moulds increased significantly in all bars and was at a level of 10^5^–10^6^ cfu/g. Similarly to the 7-day storage period, the highest number of mushrooms was detected in the bars with Psyllium fibre and quince juice and Psyllium fibre and wild rose juice (at a level of 10^6^ cfu/g).

The presence of aerobic mesophilic and spore-forming microorganisms was found in all bars (Table 4). The number of aerobic mesophilic and spore-forming microorganisms immediately after the bars were manufactured was low, at a level of 2.3–6.1 × 10^2^ cfu/g (mesophilic) and 1.1–3.4 × 10^2^ cfu/g (spore-forming). The number of spore-forming microorganisms was at a similar level after 7 and 14 days of storage of all bars (1.1–8.3 × 10^2^ cfu/g). However, the number of mesophilic bacteria increased significantly after 7 days of storage, especially in the bars with Psyllium fibre in the version with quince and wild rose juices, in which the highest number of yeasts and moulds was detected (2.5 × 10^4^ cfu/g). After 14 days of storage, the number of mesophilic microorganisms in all tested bars was at a level of 10^4^–10^5^ cfu/g.

The selection of the recipe composition and the technique of manufacturing multigrain bars are very important in shaping their microbiological safety. This aspect is as important as the nutritional value and sensory quality. The use of a high baking temperature (180 °C) for 25 min allows for a microbiologically stable product to be obtained, but it cannot be stored longer than 7 days. Due to the water content of 24–28% in baked bars and 13–18% in dried bars, and especially their high water activity (0.77–0.87), great attention should be paid to the quality of the raw materials used and the hygiene of bar production, as well as the use of appropriate packaging. These factors can guarantee high-quality bars for 7 days. The use of NFC juices from quince and blackcurrant improved the microbiological quality of the bars, especially in the recipe with blackcurrant fibre.

Probably due to the greater ability of Psyllium fibre to bind and retain water, the bar formula promoted the growth of microorganisms. On the other hand, using juices with a lower pH (mainly blackcurrant juice) contributed to limiting the growth of microorganisms. The conducted studies clearly show that the formula of multigrain bars significantly impacts their microbiological quality. Before the bars can be produced on a larger scale, research should be carried out to identify the microorganisms present and the possibility of the occurrence of mycotoxins [34]. Souiy et al. [3] assessed that sugar-free cereal bars enriched with spirulina and flavoured with neroli essential oil were suitable for consumption after 60 days of storage at room temperature (approx. 25 °C). They did not detect the presence of *E. coli*, sulphite-reducing clostridia, and pathogenic *Salmonella* spp.

## 3. Materials and Methods

### 3.1. Materials

The basic recipe of the bars included whole-grain oat flakes (Kupiec, Krzymów, Poland), pumpkin and sunflower seeds (Bakaland, Warsaw, Poland), linseed (Kresto, Skierniewice, Poland), semi-liquid barley malt extract (Ireks, Milejowice, Poland), and fibre preparations, i.e., Psyllium fibre, apple fibre, blackcurrant fibre, and cocoa fibre (coarse fibre, particle size of about 40 µm) (GreenField, Warsaw, Poland). NFC juices from Oleofarm Wrocław, Poland, from quince, chokeberry, blackcurrant, and wild rose with an extract of 12–18 °Bx were purchased from an online health food store. The nutritional content and energy value of ingredients according to the information on the manufacturers’ packaging are presented in Table 5. The recipe of the bars was determined experimentally, and the following composition was established: 14% oat flakes, 10% pumpkin seeds, 10% sunflower seeds, 10% linseed, 30% water, 20% barley malt extract, and 6% Psyllium fibre preparation.

In the modification of the recipe, Psyllium fibre was replaced with apple or cocoa and blackcurrant fibre or mixtures of apple and Psyllium fibre and apple and cocoa fibre, in a ratio of 3:2. Another modification was to replace water with NFC juices (quince, wild rose, and blackcurrant), which, in addition to binding the grain ingredients, served as an enrichment for the bars. In addition, freshly prepared juice and pomace from Ligol apples were used to assess the nutritional value of bars.

### 3.2. Technological Methods

The grain ingredients were ground in a mill (Bosch MKM6000, Gerlingen, Germany) for 20 s, so that parts of the seeds and grains were visible. To produce the bars by drying before grinding, sunflower and pumpkin seeds were roasted at 180 °C for 10 min. The obtained ingredients were mixed using a kitchen robot (Kitchen Aid, St. Joseph, MI, USA) with a planetary mixer at 4 for 3 min and left for 10 min for initial binding. Then the mass was transferred to silicone moulds with dimensions 11 × 4 × 1.2 cm, in the amount of 85 g. Their surface was levelled using a manual rolling pin.

The bars were baked in a preheated Wachtel Piccolo I oven (Pulsnitz, Germany) at 180 °C (top–bottom) for 25 min, without changing the process temperature. The second thermal method for producing bars was drying in a laboratory microwave–convection dryer (PROMIS—Tech sp. z o.o., Wrocław, Poland). The formed bars were placed on a tray and dried at 40 °C with a microwave power of 230 W. The effect of microwaves causing a temperature increase was limited by their automatic switch-off after exceeding 80 °C. The fan forced a constant air flow at a speed of 2.0 m/s. Drying was carried out for about 45 min, until the desired mass was obtained. After baking or drying, the bars were cooled at room temperature for approximately 4 h.

### 3.3. Analytical Methods

The dry matter content of the bars was determined by drying in a laboratory oven (WAMED SUP-65 WG, Warsaw, Poland) at 130 °C for 1 h [5]. Water activity was determined using AQUALAB CX-2 (Decagon Devices Inc., Pullman, WA, USA). The colour of the bars was measured using a Konica Minolta CR-300 colorimeter (Osaka, Japan) with standard observer 2°, illuminant D65, and measuring gap 8 mm, and using the CIE Lab systems.

The texture profile analysis test was performed using a TA-HD plus texturometer (Stable Micro Systems, Godalming, UK) according to the methodology described by Kowalska et al. [5]. Bars measuring 25 × 40 × 20 mm were used for the measurement. The tested samples were compressed twice to approximately 50% of the original height. The head speed was 1 mm/s. The texture parameters, such as compression work, hardness, gumminess, and chewiness, were determined.

Acrylamide content was determined by gas chromatography with mass spectrometry, GC/MS, at the Institute of Agricultural and Food Biotechnology—National Research Institute in Warsaw according to the methodology of Roszko et al. [75]. Total polyphenol content and antioxidant activity were determined by the spectrophotometric method according to the methodology described by Kowalska et al. [5].

The fatty acid composition of fat isolated from the bars was determined at the Department of Fat Technology and Food Concentrates. The fat was initially saponified according to the method of Żbikowska et al. [76]. Fatty acid composition of the fat extracted from the bars was determined by gas chromatography according to the ISO 5508:2000 standard [77]. The fatty acids were esterified according to ISO 5509:2000 [78]. Instrument: HP 6890 GC System (Agilent, Santa Clara, CA, USA), with the flame ionization detector and capillary column (60 m 9 0.25 mm ID SGE BPX 70) used. The oven temperature was from 160 to 210 °C, increasing at a rate of 2.5 °C/min. The carrier gas was helium, and the air flow rate was 30 mL/min. Detector: FID (Agilent, Santa Clara, CA, USA), 250 °C; injector: Split–Splitless, 240 °C. The software used was HP Chemstation v. 3.11. For identification of fatty acids, the Standard Supelco 37 Component FAME Mix No. 47885-U (Supelco 37, Sigma Aldrich, St. Louis, MO, USA) was used. Individual FAs are expressed as g/100 g fat. All analyses were conducted in triplicate.

Total dietary fibre content, including soluble and insoluble fibre fractions, was determined according to AOAC 991.43 and AACC 32-07.01 [79,80]. Total protein content was determined by the Kjeldahl method according to PN-EN ISO 20483:2014:02 [81]. Fat content was determined by the Soxhlet method according to PN-EN ISO 11085:2015-10 [82]. Total ash content was determined by the gravimetric method after combustion according to PN-EN ISO 2171:2023-09 [83]. Total carbohydrate content was calculated based on moisture content, total protein, total ash, fat, and total dietary fibre. The energy value of the bars was estimated by calculations based on the energy values of the ingredients, such as protein, fat, carbohydrates, and fibre [84].

The sensory evaluation was performed by a team of 30 trained individuals, aged 18 to 60, according to the methodology described by Baryłko-Pikielna and Matuszewska [85] and Handa et al. [86]. The assessors were students and Faculty of Food Technology employees with previous sensory analysis experience. The panel members were informed about the testing methodology, samples, and use of data for research purposes. All gave verbal consent to participate voluntarily in the sensory evaluation and could withdraw at any time. The assessors did not experience any danger or discomfort while testing the samples. The rights and privacy of the participants were protected by Regulation (EU) 679/2016 [87]. The study did not require formal ethical consent. The samples were randomly coded, and the assessors completed the survey anonymously. The panellists were informed about the assessed characteristics, such as appearance, smell, sweetness, taste, texture, and overall desirability, their definitions, and boundary values (Table 6). Then they were asked to mark their preferences on the structured hedonic scale with boundary terms provided in Table 6. The intensity of attributes was measured on a scale from “exceptionally undesirable” to “exceptionally desirable” (for colour, smell, texture, taste, and desirability); the results were then converted into numerical values (10 units).

Microbiological quality tests were performed immediately after production (time “0”) and after 7 and 14 days of storage in conditions simulating commercial conditions (room temperature 25 °C). Before inoculations were performed, dilutions were prepared [88] by taking 20 g of raw material or bars in aseptic conditions and introducing these weights into 180 cm^3^ of physiological saline solution. The total number of aerobic mesophilic microorganisms (PCA medium, 30 °C, 24 h) [89], the number of yeasts and moulds (DRBC medium, 28 °C, 5 days) [90], the number of coagulase-positive Staphylococci (Baird-Parker medium, 37 °C, 24 h) [91], the number of bacteria from the *Enterobacteriaceae* family (VRBG medium, 37 °C, 24 h) [92], and the number of spore-forming bacteria (PCA medium, culture from dilution after pasteurisation, 37 °C, 24 h) [93] were assessed.

### 3.4. Statistical Methods

The determination of the content of ingredients was performed in 2–3 replicates, that of colour in 5 replicates, and that of texture in 10 replicates. The influence of the type of fibre preparation used, the method of manufacturing of the bars (baking, drying), and modifications of the recipe, such as replacing water (a binding ingredient) with NFC juices, on the properties of the bars was calculated using the Statistica 13PL program, using one- or two-factor ANOVA, analysis of variance, and Tukey’s post hoc test at a significance level of 0.05.

## 4. Conclusions

1. The type of fibre (Psyllium, apple, cocoa) and replacing water as a binding component with NFC juice (quince, wild rose, blackcurrant), as well as the method of thermal processing (baking, drying), significantly affected the properties of multigrain bars. Compared to the control sample, adding fibre and NFC juices caused an approximately 10% decrease in water content and activity, especially when using cocoa fibre, blackcurrant fibre, and blackcurrant juice. Thermal processing by microwave–convection drying (40 °C/microwave power 230 W/45 min) caused a significantly greater decrease in these indicators than in baked bars (180 °C/25 min). The pH values of the bars were less diverse (4.9–6.5) and did not depend on the type of fibre. Drying and replacing water with blackcurrant juice caused a beneficial decrease in the pH of the bars, allowing for the assumption of increased microbiological safety.
2. Regardless of the processing method, the colour of the bars was shaped by both the addition of the fibre preparation and the NFC juice.
3. Compared to other fibres, Psyllium and its mixture with another fibre resulted in a significantly lower hardness and higher gumminess and chewiness. However, regardless of the type of fibre and NFC juice, and the thermal production method, the general acceptability of the bars, including texture, was positively assessed by panellists.
4. Despite the different heat treatment temperatures and chemical compositions of the bars that favour the formation of acrylamide, its content, reaching 59 µg/kg, was many times lower than the permissible level established in the Regulation of the European Commission 2017/2158/EC. The share of NFC juices significantly reduced its content in the bars, especially dried ones.
5. The bars are a source of nutrients such as carbohydrates, fat, and a high proportion of essential unsaturated fatty acids (EFAs), protein, fibre, minerals, and polyphenolic compounds, especially in the case of bars with cacao fibre. Compared to commercial bars, all bars showed a moderate caloric value (290–310 kcal/100 g).
6. The basic raw materials and those used to modify the recipe of multigrain bars were characterised by high microbiological quality.
7. Wider use of fibre preparations produced from by-products may help in the development of sustainable technologies for the production of bar snacks.

Based on this research, Psyllium fibre seems to be the most beneficial ingredient in the production of multigrain bars, through the baking and drying method, in terms of technology, health, and sensory attributes. However, apple, cocoa fibre, NFC juices, and other recipe modifications can be used depending on the needs, e.g., for people struggling with allergies and food intolerances (gluten or lactose intolerance, etc.) or looking for more natural products and sensory appeal. Using only natural ingredients can eliminate the need for sugar syrups and other undesirable ingredients in the daily diet.

## Figures and Tables

**Table 1:** Chemical composition of baked bars with Psyllium fibre and apple juice with pomace.

Apple Pomace Share [%]	Carbohydrates [%]	Fat [%]	Proteins [%]	Total Fibre [%]	Fibre Insoluble [%]	Fibre Soluble [%]	Ash [%]	TPC [mg GAE/100 g d.m.]/DPPH [µM Trolox/100 g d.m.]
0	31.22 ± 0.98	26.62 ^c^ ± 0.67	16.57 ± 0.47	9.36 ^b^ ± 0.18	7.66 ^b^ ± 0.12	1.70 ^b^ ± 0.04	3.05 ^c^ ± 0.11	334.60 ^c^ ± 11.10/23.06 ^c^ ± 1.10
2	30.45 ± 0.86	24.77 ^a^ ± 0.61	16.15 ± 0.64	9.96 ^a^ ± 0.21	6.68 ^a^ ± 0.14	3.28 ^a^ ± 0.06	2.86 ^a^ ± 0.12	286.68 ^b^ ± 7.17/90.76 ^b^ ± 19.15
6	30.36 ± 1.23	23.59 ^a^ ± 0.55	16.22 ± 0.40	9.99 ^a^ ± 0.22	6.34 ^a^ ± 0.26	3.67 ^a^ ± 0.04	2.79 ^a^ ± 0.12	264.47 ^ab^ ± 12.14/87.52 ^ab^ ± 21.04
12	30.09 ± 1.33	21.92 ^b^ ± 0.47	15.18 ± 0.34	10.47 ^a^ ± 0.32	6.37 ^a^ ± 0.23	4.08 ^a^ ± 0.04	2.60 ^b^ ± 0.12	249.52 ^a^ ± 8.81/86.16 ^a^ ± 16.20

Designations in columns a, b, and c—homogeneous groups regarding the effect of the share of pomace at *p* ≤ 0.05; no group designation when *p* > 0.05.

**Table 2:** The share of essential unsaturated fatty acids (EFAs) and saturated fatty acids in bars.

Fibre/Binder/Thermal Method		Linoleic Acid (EFAs)	Oleic Acid (EFAs)	γ-Linolenic Acid (EFAs)	Palmitic Acid (Saturated)	Stearic Acid (Saturated)	Behenic Acid (Saturated)
Ap-P	Wild rose/BAK	35.73 ^a*c*A^ ± 0.03	33.22 ^a*a*A^ ± 0.13	15.22 ^b*c*A^ ± 0.03	7.45 *^a^*^A^ ± 0.02	4.52 *^b^*^A^ ± 0.03	1.58 ^b^ ± 0.10
	Wild rose/MC	37.28 ^a*c*B^ ± 0.10	34.73 ^a*a*B^ ± 0.13	12.25 ^b*c*B^ ± 0.01	8.03 *^a^*^B^ ± 0.07	4.685 *^b^*^B^ ± 0.02	1.24 ^b^ ± 0.09
Ap-C	Quince/BAK	38.14 ^b*b*A^± 0.03	34.21 ^b*b*A^ ± 0.00	11.01 ^a*a*A^ ± 0.03	8.03 *^b^*^A^ ± 0.01	4.67 *^a^*^A^ ± 0.08	1.61 ^a^ ± 0.01
	Wild rose/BAK	37.05 ^a*b*A^ ± 0.01	33.03 ^a*b*A^ ± 0.03	13.53 *^a^*^bA^ ± 0.03	8.18 *^b^*^A^ ± 0.08	4.98 *^b^*^A^ ± 0.02	1.25 ^b^ ± 0.01
Ap-P	Quince/BAK	39.14 ^b*c*A^ ± 0.01	31.31 ^b*a*A^ ± 0.01	13.68 ^a*c*A^ ± 0.07	7.80 *^a^*^A^± 0.06	4.70 *^a^*^A^ ± 0.03	1.46 ^a^ ± 0.06
Ap-BC		37.43 ^b*a*A^ ± 0.09	33.62 ^b*c*A^ ± 0.07	12.35 ^a*b*A^ ± 0.08	8.02 *^b^*^A^ ± 0.04	4.82 ^aA^ ± 0.01	1.46 ^a^ ± 0.08

Designations of homogeneous groups: a and b—effect of the binding component; *a*, *b*, and *c*—effect of added fibre; A and B—effect of thermal method at *p* ≤ 0.05; BAK—baking; no group designation when *p* > 0.05; MC—microwave-convective drying; Ap-P, Ap-C, and Ap-BC—mixture of fibres, namely, apple and Psyllium, apple and cocoa, and apple and blackcurrant.

**Table 3:** Energy value of selected bars with fibre preparations and NFC juices [kcal/100 g].

Type of Binding Ingredient	Type of Fibre		
	Apple–Psyllium	Apple–Cacao	Blackcurrant
Water	289 ± 13	295 ± 12	290 ± 15
NFC juices: wild rose, quince, blackcurrant	302 ± 11	309 ± 12	304 ± 16

**Table 4:** Microbiological quality of baked snack bars during storage (mean values and standard deviations) *.

Fibre/NFC Juice	Yeasts and Moulds CFU/g			Aerobic Mesophilic Bacteria CFU/g			Spore-Forming Bacteria CFU/g		
	Storage Period [Days]								
	0	7	14	0	7	14	0	7	14
Psyll/Quince	nd ^A^	2.3 × 10^4 g B^ ± 3.5 × 10^1^	5.5 × 10^6 g C^ ± 1.4 × 10^4^	3.5 × 10^2 e B^ ± 1.4 × 10^1^	2.5 × 10^4 e B^ ± 0.0	1.1 × 10^5 e C^ ± 1.4 × 10^4^	1.1 × 10^2 a A^ ± 3.1 × 10^0^	1.1 × 10^2 a B^ ± 1.4 × 10^1^	2.4 × 10^2 a C^ ± 1.4 × 10^1^
Psyll/Wild Rose	nd ^A^	2.6 × 10^4 b B^ ± 1.4 × 10^1^	3.8 × 10^2 b C^ ± 2.1 × 10^1^	2.6 × 10^2 d A^ ± 1.4 × 10^1^	2.5 × 10^4 d B^ ± 1.1 × 10^2^	8.8 × 10^4 d C^ ± 2.2 × 10^3^	1.3 × 10^2 bc A^ ± 2.1 × 10^1^	2.1 × 10^2 bc B^ ± 1.0 × 10^1^	5.2 × 10^2 bc C^ ± 2.3 × 10^1^
Psyll/Blackcurrant	nd ^A^	2.1 × 10^2 f B^ ± 7.1 × 10^0^	4.5 × 10^6 f C^ ± 7.1 × 10^3^	5.9 × 10^2 c A^ ± 6.1 × 10^0^	3.7 × 10^2 c B^ ± 5.1 × 10^0^	8.7 × 10^4 c C^ ± 5.1 × 10^2^	1.3 × 10^2 d A^ ± 2.4 × 10^1^	1.4 × 10^2 d B^ ± 2.1 × 10^1^	7.1 × 10^2 d C^ ± 1.1 × 10^0^
Apple–Cacao/Quince	nd ^A^	6.0 × 10^2 d B^ ± 0.0	2.4 × 10^5 d C^ ± 7.0 × 10^3^	6.1 × 10^2 b A^ ± 1.1 × 10^1^	1.5 × 10^3 b B^ ± 1.1 × 10^2^	6.6 × 10^4 b C^ ± 2.4 × 10^3^	1.7 × 10^2 cd A^ ± 2.4 × 10^1^	2.2 × 10^2 cd B^ ± 1.1 × 10^1^	5.4 × 10^2 cd C^ ± 1.1 × 10^1^
Apple–Cacao/Wild Rose	nd ^A^	1.2 × 10^2 cd B^ ± 2.8 × 10^1^	2.3 × 10^5 cd C^ ± 1.3 × 10^4^	2.5 × 10^2 a A^ ± 5.3 × 10^0^	8.7 × 10^2 a B^ ± 2.2 × 10^1^	1.2 × 10^4 a C^ ± 2.8 × 10^3^	3.4 × 10^2 ef A^ ± 1.4 × 10^1^	3.6 × 10^2 ef B^ ± 4.1 × 10^1^	5.5 × 10^2 ef C^ ± 1.3 × 10^1^
Apple–Cacao/Blackcurrant	nd ^A^	8.2 × 10^2 c B^ ± 2.8 × 10^1^	2.1 × 10^5 c C^ ± 1.4 × 10^4^	4.4 × 10^2 a A^ ± 5.1 × 10^0^	4.6 × 10^2 a B^ ± 1.4 × 10^1^	2.4 × 10^4 a C^ ± 1.4 × 10^3^	3.3 × 10^2 b A^ ± 2.8 × 10^1^	1.8 × 10^2 b B^ ± 2.0 × 10^1^	3.3 × 10^2 b C^ ± 7.0 × 10^0^
Blackcurrant/Quince	nd ^A^	nd ^a B^	4.2 × 10^5 a C^ ± 2.1 × 10^4^	3.2 × 10^2 b A^ ± 7.1 × 10^0^	4.1 × 10^2 b B^ ± 5.3 × 10^0^	4.9 × 10^4 b C^ ± 7.1 × 10^2^	2.1 × 10^2 e A^ ± 3.1 × 10^0^	2.8 × 10^2 e B^ ± 2.2 × 10^1^	7.3 × 10^2 e C^ ± 2.2 × 10^1^
Blackcurrant/Wild Rose	nd ^A^	nd ^ab B^	5.8 × 10^5 ab C^ ± 7.1 × 10^3^	4.6 × 10^2 d A^ ± 1.1 × 10^1^	3.8 × 10^3 d B^ ± 7.0 × 10^1^	1.1 × 10^5 d C^ ± 1.4 × 10^4^	1.4 × 10^2 b A^ ± 2.0 × 10^0^	3.3 × 10^2 b B^ ± 1.4 × 10^1^	3.4 × 10^2 b C^ ± 7.1 × 10^0^
Blackcurrant/Blackcurrant	nd ^A^	2.2 × 10^2 e B^ ± 2.1 × 10^1^	5.2 × 10^5 e C^ ± 1.4 × 10^4^	2.3 × 10^2 e A^ ± 1.4 × 10^1^	1.4 × 10^3 e B^ ± 6.4 × 10^1^	1.4 × 10^5 e C^ ± 6.1 × 10^3^	2.1 × 10^2 f A^ ± 7.3 × 10^0^	2.7 × 10^2 f B^ ± 1.1 × 10^1^	8.3 × 10^2 f C^ ± 7.1 × 10^0^

Designations: * In all samples of baked bars, no presence of Staphylococci or *Enterobacteriaceae* bacteria was detected in 0.1 g during the entire storage period; nd—not detected in 0.1 g sample; homogeneous groups a, b, c, d, e, f, and g—influence of bar type; A, B, and C—influence of storage time.

**Table 5:** Nutritional content in 100 g or 100 mL portion [g] and energy value [kJ and kcal] of bar ingredients according to the manufacturers’ packaging information.

Nutritional Content/Bar Ingredients	Fat, Including Saturated Fatty Acids	Carbohydrates, Including Sugars	Protein	Fibre	Energy Value
Whole-grain oat flakes	7.6 (1.4)	69.0 (1.3)	14.0	9.0	1764/418
Pumpkin seeds	41.0 (7.3)	5.6 (1.6)	35.0	8.8	2273/548
Linseed	42.0 (3.7)	1.0 (0.5)	20.0	26.0	2119/514
Sunflower seeds	51.0 (5.4)	14.0 (2.3)	21.0	7.8	2557/618
NFC quince juice	0	11.0 (8.7)	0	-	186/44
NFC wild rose	0	10.0 (5.5)	0	3.3	209/50
NFC blackcurrant	0	8.6 (6.7)	0	0.9	162/38

**Table 6:** Definitions and boundary terms of sensory attributes.

Distinguishing Feature		Boundary Terms; Point Scores *
	Definition	
Apperance	Colour of bars (colouration)	1—exceptionally undesirable, discolouration; 10—exceptionally desirable
Smell	Intensity of the scent sensed	1—exceptionally unnoticeable, foreign; 10—exceptionally characteristic
Sweetness	Intensity of sweetness	1—exceptionally not sweet, 10—exceptionally desirable sweet
Taste	Felt after biting and chewing	1—exceptionally undesirable, foreign; 10—exceptionally desirable, characteristic
Texture	Brittleness and porosity	1—exceptionally undesirable, too hard, too compact; 10—exceptionally desirable, brittle
Desirability	The overall impression	1—exceptionally undesirable; 10—exceptionally desirable

*—visualization of how the panelist filled out the survey: 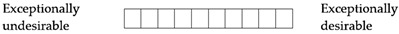.

## Data Availability

The data presented in this study are available on request from the corresponding author.
